# Human umbilical cord mesenchymal stem cell-derived neuron-like cells rescue memory deficits and reduce amyloid-beta deposition in an AβPP/PS1 transgenic mouse model

**DOI:** 10.1186/scrt227

**Published:** 2013-07-04

**Authors:** Hui Yang, ZhaoHong Xie, LiFei Wei, HongNa Yang, ShaoNan Yang, ZhengYu Zhu, Ping Wang, CuiPing Zhao, JianZhong Bi

**Affiliations:** 1 Department of Neurology Medicine, Second Hospital of Shandong University, Jinan 250033, China

**Keywords:** Alzheimer disease, AβPP/PS1 mouse, Amyloid-β peptides, Neuronal differentiation, Alternatively activated microglia, Neuroinflammation

## Abstract

**Introduction:**

Cell therapy is a potential therapeutic approach for neurodegenerative disorders, such as Alzheimer disease (AD). Neuronal differentiation of stem cells before transplantation is a promising procedure for cell therapy. However, the therapeutic impact and mechanisms of action of neuron-like cells differentiated from human umbilical cord mesenchymal stem cells in AD have not been determined.

**Methods:**

In this study, we used tricyclodecan-9-yl-xanthogenate (D609) to induce human mesenchymal stem cells isolated from Wharton jelly of the umbilical cord (HUMSCs) to differentiate into neuron-like cells (HUMSC-NCs), and transplanted the HUMSC-NCs into an AβPP/PS1 transgenic AD mouse model. The effects of HUMSC-NC transplantation on the cognitive function, synapsin I level, amyloid β-peptides (Aβ) deposition, and microglial function of the mice were investigated.

**Results:**

We found that transplantation of HUMSC-NCs into AβPP/PS1 mice improved the cognitive function, increased synapsin I level, and significantly reduced Aβ deposition in the mice. The beneficial effects were associated with “alternatively activated” microglia (M2-like microglia). In the mice transplanted with HUMSC-NCs, M2-like microglial activation was significantly increased, and the expression of antiinflammatory cytokine associated with M2-like microglia, interleukin-4 (IL-4), was also increased, whereas the expression of proinflammatory cytokines associated with classic microglia (M1-like microglia), including interleukin-1β (IL-1β) and tumor necrosis factor-α (TNF-α), was significantly reduced. Moreover, the expression of Aβ-degrading factors, insulin-degrading enzyme (IDE) and neprilysin (NEP), was increased substantially in the mice treated with HUMSC-NCs.

**Conclusions:**

HUMSC-NC transplantation decreased Aβ deposition and improved memory in AβPP/PS1 mice by a mechanism associated with activating M2-like microglia and modulating neuroinflammation. Transplantation of neuron-like cells differentiated from mesenchymal stem cells might be a promising cell therapy for Alzheimer disease.

## Introduction

Alzheimer disease (AD) is an age-related progressive neurodegenerative disorder. The major symptoms of AD include memory loss and severe cognitive decline. Pathology of the AD brain is characterized by amyloid plaques, neurofibrillary tangles, and neuronal loss. Elevated amyloid β-peptide (Aβ) deposition is the key pathogenic factor for AD and the main cause for neuronal loss in AD [1]. Thus, promising therapeutic strategies for AD aim to prevent, reverse, and reduce Aβ deposition [2].

Cell therapy is a potential therapeutic approach for neurodegenerative disorders, such as AD [3,4]. It has been found that transplantation of cells isolated from human umbilical cord, mesenchymal stem cells (MSCs), or neural progenitor cells improves neuropathology in animal models of AD through modulation of neuroinflammation [5-8]. Recently, a number of studies demonstrated that transplantation of neuronal cells induced from MSCs produces beneficial effects in neurodegenerative diseases and spinal cord injury [9-11]. It has been shown that neuronal cells differentiated from human MSCs are more resistant to Aβ_42_ oligomer-induced cytotoxicity than are undifferentiated cells [12]. Thus, neuronal differentiation before transplantation could yield better efficacy than transplantation of undifferentiated MSCs in clinical application.

The mechanism underlying the beneficial effects of stem cell transplantation on neurodegenerative diseases has been found to be associated with microglial function in brain. Microglia, the mononuclear phagocytes of brain, accumulate in senile plaques in AD patients and in animal models of AD. It has been shown that microglia release proinflammatory cytokines, such as tumor necrosis factor-α (TNF-α), interferon-γ (IFN-γ), interleukin-1β (IL-1β), and NO, causing neurodegeneration [13]. In contrast, growing evidence has also supported that “alternatively activated” microglia (M2-like microglia) play protective roles in AD by phagocytizing Aβ and secreting neurotrophic cytokines [14]. Multiple studies demonstrated that intracerebral transplantation of MSCs increases M2-like microglial activation, regulates neuroinflammation, and reduces Aβ deposits in AD mouse models [8,9,15].

In this study, we used tricyclodecan-9-yl-xanthogenate (D609) to induce HUMSCs to differentiate into neuron-like cells (HUMSC-NCs) and transplanted the HUMSC-NCs into an AβPPswe/PS1dE9 mouse model of AD. We found that transplantation of HUMSC-NCs reduced Aβ deposition in the mouse and improved the mouse’s cognitive function. The beneficial effects might be associated with M2-like microglial activation and modulation of neuroinflammation by HUMSC-NC transplantation.

## Materials and methods

### Animals

The heterozygous AβPPswe/PS1dE9 double-transgenic mouse with C57BL/6 background was used in this study. The mouse harbors the mutant human genes APPswe (Swedish mutations *K594N/M595L*) and presenilin-1 with the exon-9 deletion (*PS1-dE9*) under the control of mouse prion protein promoter. This type of transgenic mouse has been used widely in the study of AD [16,17]. The mouse shows typical characteristics of AD and exhibits an early appearance of amyloid plaques deposition and increased proinflammatory microglial activation [17,18]. Because of the gender-specific differences in the progression of amyloid plaque deposition, we used only male mice in this study. The mice were obtained from Beijing HFK Bio-Technology Co., Ltd., Institute of Laboratory Animal Science, Chinese Academy of Medical Science (Beijing, China), and housed in temperature- and humidity-controlled rooms on a 12 hour/12 hour light/dark cycle. Breeder mice carrying the transgenes are backcrossed to the C57BL/6 strain for six to seven generations to obtain mice used in research. The mice are genotyped to confirm the presence of the mutant genes by polymerase chain reaction (PCR) amplification of genomic DNA extracted from tail clippings before the mice are sold to research laboratories. All the procedures described in this study were in accordance with the Ethical Committee for Animal Experiments of Shandong University.

### HUMSC isolation and *in vitro* culture

Human umbilical cords were obtained from full-term deliveries with the informed consent from parents after caesarian section. The procedure for collecting tissues was approved by the ethical committee of the Second Hospital of Shandong University. The procedure was based on the previous description by Huang *et al.*[19]. After arteries and veins were removed, the remaining tissue, Wharton jelly, was cut into 0.5- to 1-cm^3^ pieces and suspended in Dulbecco modified Eagle low-glucose media (DMEM-LG; Gibco, Grand Island, NY, USA) containing 10% fetal bovine serum (FBS; Gibco), 100 mg/ml penicillin, and 100 mg/ml streptomycin. The tissue was left undisturbed for 7 days in a 37°C humidified incubator with 5% CO_2_ to allow cells to migrate from the explants. Culture media was replaced every 3 days. The cells were passaged by using 0.25% trypsin-EDTA solution when they reached 80% to 90% confluence. The cells were analyzed with flow cytometry to confirm the immune-phenotype of HUMSCs according to the previous report [20]. The cells used in this study were positive for CD73, CD90, and CD105, but negative for CD34, CD45, and HLA-DR, consistent with HUMSC characteristics.

### Induction of neuron-like cells from HUMSCs

After HUMSCs were passaged 2 to 6 times and reached subconfluence, the cells were washed twice with basal DMEM media (without FBS) and divided into two groups. In the control group, the cells were cultured in basal DMEM medium (without FBS); in the D609 treatment group, the cells were cultured in basal media containing 60 μg/ml D609 (J&K, Beijing, China) to induce neuronal differentiation. D609 was prepared freshly in deionized water. We determined the optimal working concentration of D609 by performing a dose response of HUMSCs to 2 to 100 μg/ml of D609, and we found that 60 μg/ml D609 was the optimal concentration for neuronal differentiation (data not shown). The morphology of cells was observed under a phase-contrast microscope.

### Immunocytochemistry

To determine the expression of neuronal cell markers, HUMSCs grown on glass coverslips were treated with D609 or basal medium for 6 hours. The cells were fixed in 4% paraformaldehyde in phosphate-buffered saline (PBS) for 20 minutes and then washed 3 times with PBS. The cells were blocked with normal goat serum for 20 minutes at room temperature and then incubated in the following primary antibodies: neuron-specific enolase (NSE, rabbit IgG, 1:200, Abcam, Cambridge, MA, USA), microtubule-associated protein 2 (MAP2, rabbit IgG, 1:200, Abcam), and glial fibrillary acidic protein (GFAP; rabbit IgG, 1:200, Sigma, St Louis, MO, USA). The cells were then washed in PBS and incubated with the secondary antibodies (goat anti-rabbit IgG-TRITC) for 1 hour at room temperature. After washing with PBS, the cells were observed under a fluorescence microscope (Olympus 1 × 71S1F-3, Tokyo, Japan). The differentiation rate of HUMSCs was calculated according to the following formula: The differentiation rate (percentage) = (the number of NSE or MAP2 positive cells/the total number of cells) ×100. For each sample, 10 randomly selected visual fields were used for the calculation. The results presented are the mean ± SEM from three independent experiments.

### Transplantation of HUMSC-NCs in AβPP/PS1 double-transgenic mice

HUMSC-NC suspension, HUMSC suspension, or PBS was injected into 6-month-old male AβPP/PS1 transgenic mice for only one time. Before the injection, the cell suspension was washed with PBS for 3 times to remove drug and serum. Animals were anesthetized with 10% chloral hydrate and placed in a small animal stereotaxic apparatus with a microinjector unit. Either cell suspension or PBS was injected into mouse bilateral hippocampus at the following coordinates: 1.6 mm posterior to the bregma, 1.7 mm bilateral to the midline, and 2.5 mm ventral to the skull surface. Cell suspension was injected with a 25-μl syringe. Then 2 μl of cell suspension (approximately 5 × 10^4^ cells) was injected into the hippocampus bilaterally. Cell suspension or PBS was delivered at a rate of 0.3 μl/min. After injection, the needle was left in place for 5 minutes before being retracted.

### Behavior test

Three weeks after transplantation, we used the modified Morris water-maze test to assess spatial memory performance [21]. The procedure consisted of 1 day of adapting tests without platform and 5 days of hidden-platform tests, plus a spatial probe test 24 hours after the last hidden-platform test; 15 mice were used in each group. The detailed method was described in our previous report [22]. In brief, in the spatial-acquisition tests, mice were released into the pool and given 60 seconds to find the hidden platform. If a mouse did not find the platform within 60 seconds, it was guided to the platform. Animals were given four trials per day. The distal starting positions were semirandomly selected. For basic acquisition training, the platform was located in the southwest quadrant. The starting positions were north, east, southeast, and northwest. The time to find the platform was recorded as the latency for each trial. A single probe trial, in which the platform was removed, was performed after the hidden-platform task had been completed. Mice were placed in a novel start position (northeast) in the maze, and each mouse was allowed to swim for 60 seconds. The data were analyzed with multivariate analysis of variance (ANOVA). Mouse behavior was observed blindly. Observers were kept ignorant of the treatment given to each animal group.

### Thioflavin S staining and immunohistochemical staining

AβPP/PS1 mice were killed after the behavior test. The mice were anesthetized with chloral hydrate, and then were immediately cardiac perfused with 0.9% saline solution followed by 4% paraformaldehyde in 0.1 *M* PBS (pH 7.4). After the perfusion, the brains of the mice were excised and postfixed overnight at 4°C. The brain tissue was then incubated in 30% sucrose at 4°C until equilibration (six mice per group). Then 30- or 10-μm coronal sections were cut with a freezing microtome (Leica CM1850, Leica Microsystems, Heidelberg Germany) and stored at −20°C.

Thioflavin S staining was performed on floating sections (30-μm thickness). Brain sections were incubated in 0.5% thioflavin S (Sigma-Aldrich, USA) dissolved in 50% ethanol for 5 minutes, and then washed twice with 50% ethanol for 5 minutes each time. The brain sections were washed once with tap water for 5 minutes, and then mounted with mounting medium [8]. The green fluorescence-stained plaques were observed under a fluorescence microscope. Frontal cortex, cingulate, and hippocampus were examined for amyloid load. According to the previous report [23], these regions have plaque prevalence in AD patients and are involved in memory functions.

For immunohistochemical staining, frontal cortex and hippocampus sections (10-μm thickness) were incubated with primary antibody at 4°C overnight. The following primary antibodies were used: Iba-1 (rabbit IgG, 1:500, Wako, Richmond, VA, USA); IL-4 (goat IgG, 1:200, Santa Cruz, Dallas, Texas, USA); TNF-α (goat IgG, 1:200, Santa Cruz); AMCase, (goat IgG, 1:200, Santa Cruz). Fluorescent dye-conjugated secondary antibodies (IgG-FITC or IgG-TRITC) were used to visualize the staining. To quantify the IHC staining, 10 serial cortex and hippocampal sections (at an interval of 50 μm for each section) from each animal (*n* = 6 for each group) were used to quantify each parameter. The staining was analyzed with the image-analyzing system, Image Pro Plus 6 (Media Cybernetics, Rockville, MD, USA).

### Western blot analysis

Cerebral cortex and hippocampus from one hemisphere were isolated from AβPP/PS1 mice of each group after the behavior test (six mice per group). Brain tissue samples were snap frozen and stored at −80°C for future experiment. The brain samples were homogenized in ice-cold RIPA lysing buffer (Beyotime, Shanghai, China). The homogenized samples were centrifuged at 12,000 *g* for 20 minutes at 4°C. The supernatant was collected for Western blot. The proteins were separated with SDS-PAGE and transferred to PVDF membranes. The membranes were blocked with 5% nonfat dry milk in TBST for 1 hour and incubated with primary antibodies overnight at 4°C. The following primary antibodies were used: Synapsin I (rabbit IgG, 1:1,000, Abcam), NEP (goat IgG, 0.2 μg/ml, R&D, Minneapolis, MN, USA); IDE (rabbit IgG, 1:5,000, Abcam); and β-actin (mouse IgG, 1:400, Santa Cruz) [22]. The following secondary antibodies were used: goat anti-mouse IgG/HRP (1:5,000, Golden Bridge International, Beijing, China); goat anti-rabbit IgG/HRP (1:5,000, Golden Bridge International); rabbit anti-goat IgG/HRP (1:5,000, Golden Bridge International). The intensity of the bands was quantified by using Image J software developed by Wayne Rasband, National Institutes of Health, Bethesda, MD, USA.

### Aβ ELISA

Aβ_40_ and Aβ_42_ enzyme-linked immunosorbent assays (ELISAs) were performed by using ELISA kits (Invitrogen, Carlsbad, CA, USA). Aβ standards (1 to 40 or 1 to 42 aa) were used according to the manufacturer’s manual. Hippocampus and cortex from one hemisphere were homogenized in eight volumes of ice-cold guanidine buffer (5.0 *M* guanidine-HCl 50 m*M* Tris–HCl, pH 8.0) (six mice per group). The homogenate was incubated at room temperature for 4 hours and diluted at 1:20 with ice-cold reaction buffer BSAT-DPBS (Dulbecco phosphate-buffered saline, with 5% BSA, 0.03% Tween-20, 0.2 g/L KCl, 0.2 g/L KH_2_PO_4_, 8.0 g/L NaCl, 1.150 g/L Na_2_HPO_4_, pH7.4) containing 1 × Protease Inhibitor Cocktail (PMSF, aprotinin, leupeptin, EDTA, pepstatin A, NaF, and NaVO_3_), and then was centrifuged at 15,000 *g* at 4°C for 20 minutes. The supernatant was used for ELISA assay.

### Quantitative real-time PCR

Total RNA from cortex and hippocampus of one hemisphere was extracted by using Trizol (Invitrogen) according to the manufacturer’s protocol (six mice per group); 1 μg total RNA was used for reverse transcription in a final volume of 20 μl. Reverse transcription was performed according to the manufacturer’s protocol (DRR047A, Takara, Otsu, Shiga, Japan). Then 2 μl cDNA was used for real-time PCR with the SYBR Premix Ex Taq (DRR041A, Takara, Japan). Quantitative real-time PCR was performed under the following conditions: 95°C for 30 seconds, 95°C for 5 seconds, 60°C for 34 seconds, and 40 cycles. All PCR reactions were performed in triplicate. The fold changes of target genes were calculated by using the delta delta cycle threshold (two delta delta CT) method [24]. GAPDH was used as the reference gene. The PCR primers used in the study were reported previously [8].

### Statistical analysis

All data were presented as mean ± standard error of the mean (SEM). Differences were analyzed with ANOVA. A value of *P* < 0.05 was considered statistically significant. In two-variable experiments, two-way ANOVA followed by Bonferroni *post hoc* tests were used to analyze the significance of differences between groups. One-way ANOVA was used to analyze one-variable experiments with three groups. The Student *t* test was used to compare two groups. Data were analyzed with the software SPSS 16.0 (SPSS Inc., Chicago, IL, USA).

## Results

### D609 induces neuron-like differentiation of HUMSCs

**Figure 1:**
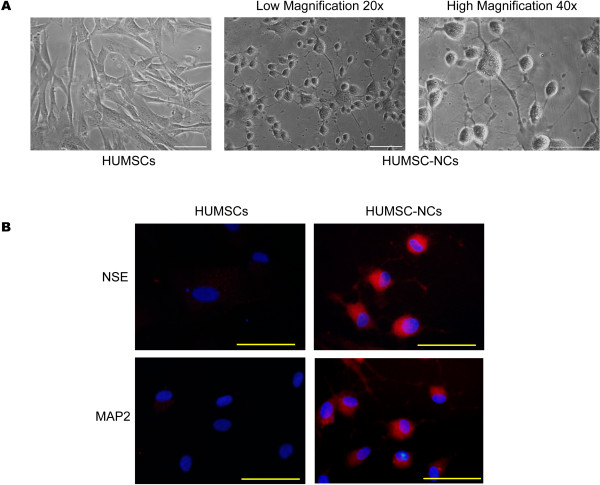
**Neuron-like cell differentiation from HUMSCs by D609 induction. (A)** Representative images of HUMSCs and HUMSC-NCs obtained by an inverted phase-contrast microscopy. Scale bar, 50 μm. **(B)** Immunocytochemistry staining for neuronal markers NSE (red) and MAP2 (red) in HUMSCs and HUMSC-NCs. Cells were fixed and stained with rabbit anti-human NSE IgG (1:200) or rabbit anti-human MAP2 IgG (1:200). Fluorescent dye-conjugated secondary antibody, goat anti-rabbit IgG-TRITC, was used to visualize the cells. Nuclei were stained with DAPI (blue). The images were captured with a camera system connected to a fluorescence microscope (Olympus 1x71S1F-3, Japan). Scale bar, 50 μm.

Wang and colleagues found that D609 can induce rat MSCs and human bone marrow stem cells to differentiate into neuron-like cells within 6 hours. Thus, we examined the morphologic change and the expression of neuronal markers of HUMSCs after 6-hour treatment with D609. HUMSCs appeared as large and flat cells in the absence of D609 treatment. After 6 hours of D609 treatment, most of the cells exhibited a typical neuron-like morphology characterized by a multipolar cell body with peripheral extended processes, and the processes formed a connected network; no obvious morphologic change was found in the control group (HUMSCs, Figure 1A). Immunocytochemistry analysis showed that HUMSC-NCs expressed high levels of the neuronal cell markers, neuron-specific enolase (NSE) and microtubule-associated protein 2 (MAP2) (Figure 1B). The percentage of cells expressing NSE or MAP2 was 86.42% ± 6.787% or 82.39% ± 5.644% in the D609-treated group. However, in the control groups, HUMSCs expressed NSE or MAP2 weakly (Figure 1B). To examine further the quality and quantity of the neuronal differentiation, we examined the expressions of glial fibrillary acidic protein (GFAP), a marker for glial cells, in the D609-induced cells. We did not find GFAP expression in the differentiated cells, indicating that D609 induces HUMSCs to differentiate into neuron-like cells but not glial cells (see Additional file 1: Figure S1).

### HUMSC-NC transplantation improves spatial learning and alleviates memory impairments in AβPP/PS1 mice

**Figure 2:**
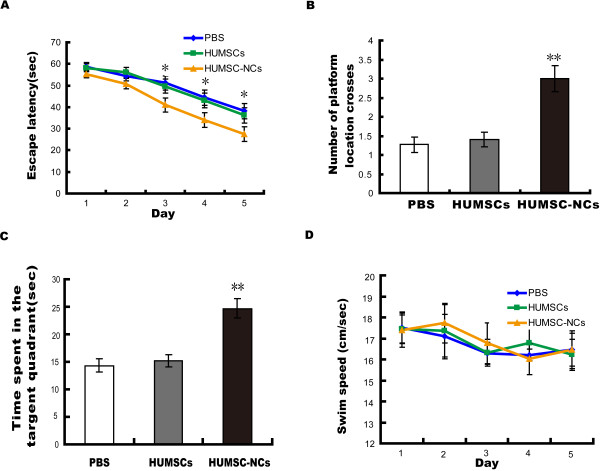
**HUMSC-NC transplantation improved spatial learning and alleviated memory impairments in AβPP/PS1 mice. (A)** HUMSC-NC transplantation improved spatial learning in AD mice. Spatial learning was measured as escape latencies per day. Fifteen mice were used in each group. **(B, C)** HUMSC-NC transplantation restored spatial memory in the AD mice. Mouse spatial memory was evaluated by the number of platform location crosses **(B)** and the time spent in the target quadrant **(C)**. **(D)** Swimming speed was not significantly different between groups. Data are presented as mean ± SEM; *P < 0.05, **P < 0.01, HUMSC-NC-treated group versus PBS-treated group.

To determine the effect of HUMSC-NC transplantation on the behavior of AβPP/PS1 mice, we used a Morris water maze to examine the spatial learning and memory of the mice 3 weeks after they received HUMSC-NC, HUMSC, or PBS injection. No difference in behavioral performance was detected in the HUMSC-treated mice compared with the PBS-treated mice. However, the mice treated with HUMSC-NCs performed significantly better in the water-maze test than did the mice treated with PBS. The mean escape latency of the mice treated with HUMSC-NCs was significantly shorter than that of the mice treated with PBS (2A). We evaluated mouse spatial memory by performing probe trials 24 hours after the last training session. The number of platform location crosses and the time spent in the target quadrant of the mice treated with HUMSC-NCs was significantly higher and longer than were those of the mice treated with PBS (Figure 2B, C). The swimming speed was not significantly different among the three groups (Figure 2D), suggesting that the improved behavioral performance of the mice treated with HUMSC-NCs was caused by cognitive processes but not noncognitive components of behavior. Thus, our data suggest that single intracerebral injection of HUMSC-NCs alleviates learning deficits and memory impairments in AD mice, whereas single injection of HUMSCs does not.

### HUMSC-NC transplantation increases synapsin I level

**Figure 3:**
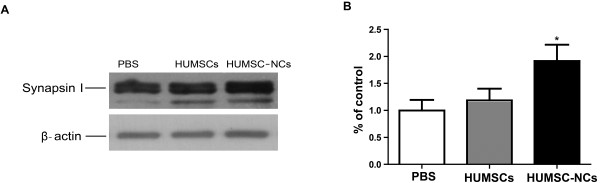
**HUMSC-NC transplantation increased synapsin I level. (A)** Representative Western blots for synapsin I protein expression in hippocampus. **(B)** Quantification of the Western blot for synapsin I. The densitometry of synapsin I bands was first normalized to the loading control β-actin. The percentage of the expressions of synapsin I in the HUMSC- or HUMSC-NC-treated group relative to that in the PBS-treated group was then calculated. The scanned image of Western blot was analyzed with the software Image J. Data were presented as mean ± SEM. *P < 0.05, HUMSC-NC-treated group versus PBS-treated group.

The cognitive impairment in AD is associated mostly with synaptic loss and dysfunction. Synapsin I has been shown to play a key role in functional maturation of synapse and neurotransmitter release [25]. We then compared the expression of synapsin 1 protein in the hippocampus between HUMSC-NC-transplanted and PBS-treated mice. We found that synapsin I protein expression in hippocampus was increased significantly in HUMSC-NC-transplanted mice compared with that in PBS-treated mice (Figure 3), suggesting that the cognitive improvement in HUMSC-NC-treated mice might be associated with the upregulation of synapsin 1.

### HUMSC-NC transplantation significantly reduces Aβ deposition and decreases soluble Aβ levels

**Figure 4:**
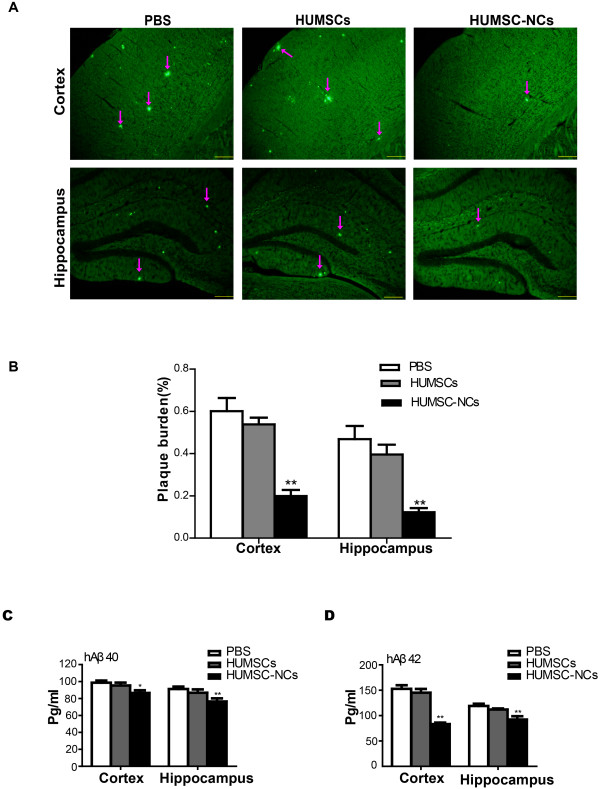
**HUMSC-NC transplantation reduced Aβ deposition and soluble Aβ levels. (A)** Thioflavin S staining for senile plaques of cerebral cortex and hippocampus in the mice. The staining was performed according to the description in the Methods section. Images were captured with a camera system connected to a fluorescence microscope (Olympus 1×71S1F-3, Japan). Scale bar, 200 μm. **(B)** Quantification of thioflavin S staining. The plaque burden was calculated as the percentage of thioflavin S staining area over the total area (*n* = 6 in each group). Image Pro Plus 6 (Media Cybernetics) was used to analyze the images. **(C, D)** ELISA for soluble Aβ_40_**(C)** and Aβ_42_**(D)** in the cortex and hippocampus. Mouse brain tissue was homogenized, and soluble protein was extracted. Aβ ELISA kits (Invitrogen) was used to determine the levels of Aβ_40_ and Aβ_42_ (*n* = 6 in each group). Data are presented as mean ± SEM; *P < 0.05; **P < 0.01; HUMSC-NC-treated group versus PBS-treated group.

Aβ deposition is the key pathogenic factor for AD and causes cognitive deficits. We investigated the effect of HUMCS-NC transplantation on Aβ deposition in AD mice. We used thioflavin S staining to determine Aβ deposition. We found that Aβ deposition in both the cortex and hippocampus of the mice treated with HUMSC-NCs was dramatically reduced compared with that of the mice treated with PBS (Figure 4A, B). Because Aβ can be released by cleavage of a99-residue C-terminal fragment (CTF), we measured the CTF level in the cortex or hippocampus of the mice with Western blot. No difference in the CTF level was found among the three groups (data not shown), indicating that HUMSC-NC transplantation does not affect Aβ production. Further to confirm the reduction of Aβ deposition by HUMSC-NC transplantation, we performed ELISA to quantify soluble Aβ_40_ and Aβ_42_ levels in the mice. We found that both Aβ_40_ and Aβ_42_ levels in the mice treated with HUMSC-NCs were significantly decreased compared with those in the mice treated with PBS (Figure 4C, D). Our results suggest that HUMSC-NC transplantation significantly reduces Aβ deposition in the AD mice.

### HUMSC-NC transplantation stimulates microglial activation in AβPP/PS1 mice

**Figure 5:**
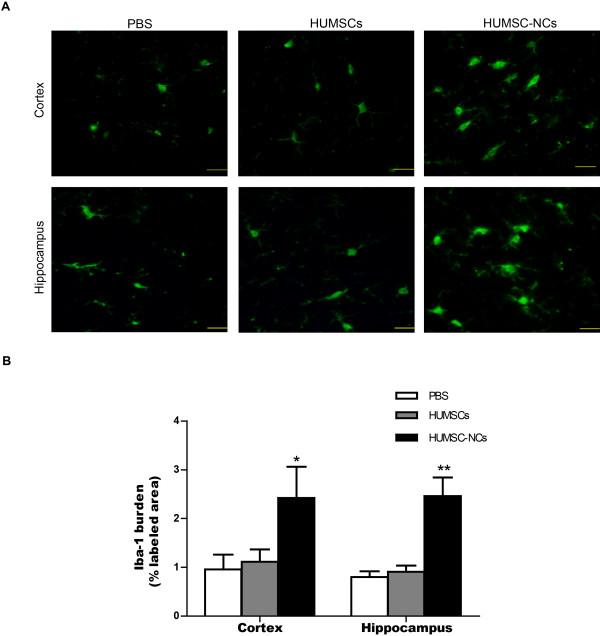
**HUMSC-NC transplantation increased microglial activation. (A)** Immunofluorescence staining for microglia with anti-Iba1 in both the hippocampus and cortex. The staining was performed as described in the Methods section. The processed tissue section was stained with rabbit anti-mouse Iba-1 IgG (1:500, Wako), and then visualized with FITC-conjugated secondary antibody. Scale bar represents 20 μm. **(B)** Quantification of the staining images. Iba-1 burden was calculated as the percentage of Iba-1-positive area over the total area. The image quantification was performed by using the software Image Pro Plus 6 (Media Cybernetics). Data are presented as mean ± SEM. *P < 0.05; **P < 0.01; HUMSC-NC-treated group versus PBS-treated group.

We then further investigated the mechanism by which HUMSC-NC transplantation reduces Aβ deposition. In our study, we discovered that HUMSC-NC transplantation significantly increased the number of activated microglia in the cortex and hippocampus of the mice compared with PBS injection (Figure 5A, B). The Iba1 staining showed that the microglia in the mice treated with HUMSC-NCs displayed an activated microglial morphology characterized by large cell body and thick processes (Figure 5A). The density of the activated microglia reached the highest around the plaques, corpus callosum, hippocampus, and cortex area near the injection site (data not show). Microglial activation was not observed in the mice treated either with HUMSCs or with PBS injection. These data indicate that HUMSC-NC transplantation stimulates microglial activation in AβPP/PS1 mice.

### HUMSC-NC transplantation induces alternative microglial activation and modulates neuroinflammation

**Figure 6:**
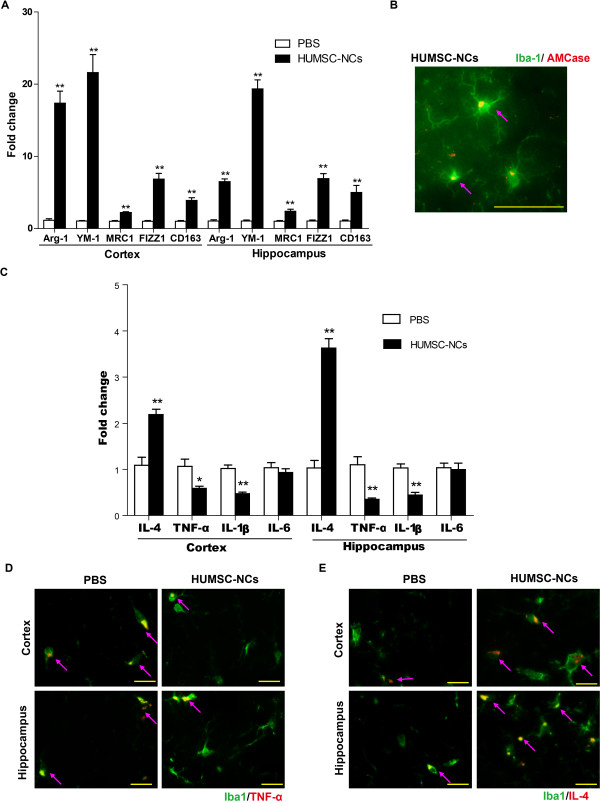
**HUMSC-NC transplantation induced M2-like microglial activation and modulated neuroinflammation. (A)** mRNA levels of Arg-1, YM-1, MRC1, FIZZ1, and CD163 in the cortex or hippocampus were determined by quantitative RT-PCR. GAPDH was used as the reference gene. (*n* = 6 in each group). **(B)** Immunofluorescence staining for AMCase and Iba-1. Tissue sections were processed and stained with rabbit anti mouse Iba-1 IgG (1:500; Wako) and goat anti-mouse AMCase IgG (1:200, Santa Cruz). FITC- or TRITC-conjugated secondary antibodies (1:200) were used to visualize the staining. Scale bar represents 50 μm. **(C)** mRNA levels of IL-4, TNF-α, IL-1β, and IL-6 in the cortex or hippocampus were measured with quantitative RT-PCR (*n* = 6 in each group). **(D)** Double immunofluorescence staining for TNF-α and Iba-1 in the cortex and hippocampus area. **(E)** Double immunofluorescence staining for IL-4 and Iba-1 in the cortex and hippocampus area. The images were merged from individual images with different staining. Images were captured by a camera system connected to the fluorescence microscope (Nikon Eclipse 90i LH-M100CB-1). Scale bar represents 20 μm. Data are presented as mean ± SEM. *P < 0.05; **P < 0.01; HUMSC-NC-treated group versus PBS-treated group.

Activated microglia can be either neuroprotective or neurodestructive, depending on the phase of activation. Alternatively, activated microglia (M2-like microglia) have been found to protect neurons by increasing Aβ clearance and reducing neuroinflammation. Thus, we used the following markers for M2-like microglia: Chitinase 3-like 3 (YM-1), arginase-1 (Arg-1), AMCase, mannose receptors C type 1 (MRC1), found in inflammatory zone 1 (FIZZ1), and haptoglobin/hemoglobin scavenger receptor (CD163) to examine whether the activated microglia induced by HUMSC-NC transplantation were M2-like microglia. Our qRT-PCR assay showed that the gene-expression levels of YM-1, Arg-1, MRC1, FIZZ1, and CD163 in both the cortex and hippocampus of the mice treated with HUMSC-NCs were increased substantially, compared with those in the mice treated with PBS (Figure 6A). Our immunofluorescence staining also demonstrated that AMCase expression was colocalized with Iba-1expression in microglia (Figure 6B), indicating that the activated microglia induced by HUMSC-NC transplantation have M2-like microglial characteristics. Our results suggest that HUMSC-NC transplantation induces M2-like microglial activation in AD mice.

AβPPswe/PS1dE mice have significantly higher levels of proinflammatory cytokines, including IL-1β, IL-6, and TNF-α, than do wild-type mice [18]. Reducing these cytokines has been shown to prevent neuronal dysfunction in AD [26]. We examined whether the beneficial effects of HUMSC-NC transplantation were associated with a reduction of the proinflammatory factors IL-1β, TNF-α, and IL-6. Our RT-PCR result showed that the mRNA levels of IL-1β and TNF-α were significantly reduced in the cortex and hippocampus of the mice treated with HUMSC-NCs compared with those in the mice treated with PBS (Figure 6C), whereas the IL-6 level was not different between the two groups. M2-like microglial activation has been shown to be associated with upregulation of the antiinflammatory cytokine, IL-4 [27]. Our RT-PCR result showed that the mRNA level of IL-4 in the cortex and hippocampus of the mice treated with HUMSC-NCs was significantly increased compared with that of the control (Figure 6C).

To determine further whether the downregulation of the proinflammatory cytokine TNF-α and the upregulation of the antiinflammatory cytokine IL-4 were related to the activated microglia in the mice treated with HUMSC-NCs, we performed double immunofluorescence staining for TNF-α and Iba-1, or IL-4 and Iba-1. Significant increase of IL-4 expression and decrease of TNF-α expression were detected in the Iba-1-positive cells in the cortex and hippocampus of the mice treated with HUMSC-NCs compared with those in the control (Figure 6D, E). This result suggests that M2-like microglial activation is associated with the modulation of neuroinflammation in the AD mice.

### The expression of Aβ-degrading factors is stimulated by HUMSC-NC transplantation

**Figure 7:**
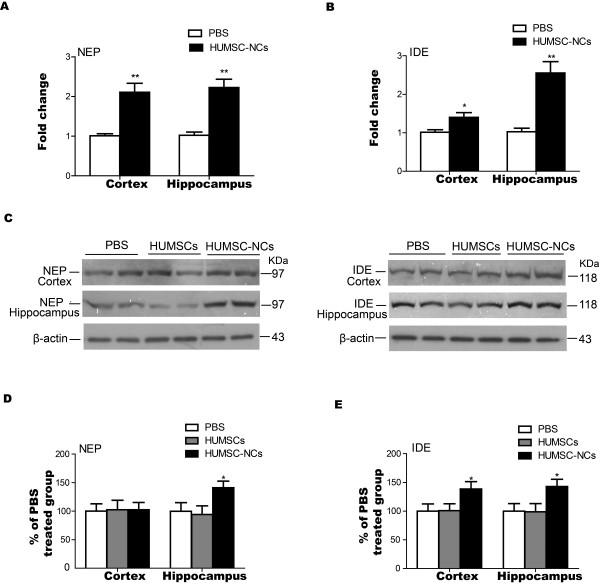
**HUMSC-NC transplantation increased the expression of Aβ-degrading enzymes NEP and IDE. (A, B)** The mRNA levels of NEP **(A)** and IDE **(B)** in the cortex or hippocampus were determined with RT-PCR. Both NEP and IDE mRNA levels were increased significantly in the cortex and hippocampus of HUMSC-NC-treated mice. **(C)** Representative Western blots for NEP and IDE protein expression in the cortex or hippocampus: 100 μg of protein was load in each lane. Duplicate samples of each group were loaded into the gel. **(D, E)** Quantification of the Western blot for NEP **(D)** and IDE **(E)**. The densitometry of NEP and IDE bands were first normalized to the loading control β-actin. The percentage of the expressions of NEP and IDE in the HUMSC- or HUMSC-NC-treated group relative to that in the PBS-treated group was then calculated. The scanned image of Western blot was analyzed with the software Image J. Data were presented as mean ± SEM. *P < 0.05, HUMSC-NC-treated group versus PBS-treated group.

Neprilysin (NEP) and insulin-degrading enzyme (IDE) are the most important Aβ-degrading enzymes. Our RT-PCR result demonstrated that the mRNA levels of NEP and IDE in the mice treated with HUMSC-NCs were significantly higher than those in the mice treated with PBS (Figure 7A, B). IDE protein level in both the cortex and hippocampus was also significantly increased by HUMSC-NC transplantation (Figure 7C, E), whereas NEP protein level was significantly elevated in the hippocampus but not in the cortex of the mice treated with HUMSC-NCs (Figure 7C, D) compared with that in the control. Our results indicate that HUMSC-NC transplantation reduces Aβ deposition in AD mice by increasing Aβ removal.

## Discussion

In this study, we used D609 to induce HUMSCs to differentiate into HUMSC-NCs and transplanted the HUMSC-NCs into AβPP/PS1 mice. We found that HUMSC-NC transplantation reduced Aβ deposition and alleviated cognitive decline through a mechanism associated with activating M2-like microglia and upregulating neuroprotective cytokines.

D609 has been shown to increase markedly the expression level and activity of molecules associated with the oxidative state of MSCs, including reactive oxygen species (ROS), NADPH oxidase, and Rb protein [28]. The possible mechanism underlying D609-induced neuronal differentiation of HUMSCs might be associated with the changes of these molecules.

The mice were not provided immunosuppression. HUMSCs have been found to be able to escape immune surveillance, possibly because of the absence of the antigen major histocompatibility complex II (MHC-II) and the co-stimulatory surface antigens CD40, CD80, and CD86 [29-31]. It has also been shown that neuron-like cells differentiated from human MSCs do not express CD40 and CD86 [32]. These findings suggest that both HUMSCs and the neuron-like cells differentiated from human MSCs might have low immunogenicity and might be well tolerated in xenotransplantation without immunosuppression. Consistent with our findings, a number of studies have demonstrated that transplantation of human MSCs or neuron-like cells differentiated from MSCs without immunosuppression produces beneficial effects in animal models of AD, Parkinson disease, traumatic brain injury, and stroke [10,33-36].

The beneficial effects produced by HUMSC-NC transplantation might not directly come from the HUMSC-NCs, but instead could be indirect effects caused by the transplanted cells. We found injected cells in the hippocampus and cortex of the mice 1 week after the transplantation (data not shown); however, 1 month after the transplantation, few transplanted cells were detected, both in the mice treated with HUMSCs and in the mice treated with HUMSC-NCs. Similar observations have been reported by other research groups. Hong *et al.*[33] and Parr *et al.*[37] showed that only small numbers of transplanted stem cells or induced neuron-like cells engraft into injured tissues, and only a few grafted cells survive. It has been proposed that the beneficial outcome of cell transplantation in neurodegenerative diseases could result from the paracrine factors induced by cell transplantation and be sustained by the paracrine factors even after transplanted cells die [7,38]. Our results supported such hypotheses. We found that the expression of antiinflammatory cytokine IL-4 was significantly increased by HUMSC-NC transplantation, which could lead to the induction of M2-like microglial activation and Aβ removal in the AD mice.

NEP and IDE are the most important Aβ-degrading enzymes. We found that HUMSC-NC transplantation significantly increased IDE and NEP expression in the AD mice. Deletion of the *NEP* or *IDE* gene in mice increases cerebral Aβ accumulation, whereas overexpression of NEP and IDE reduces Aβ levels [39-41]. Multiple studies showed that microglia secretes IDE and NEP [8,42,43]. Antiinflammatory cytokine IL-4 has also been found to increase IDE and NEP levels, both *in vitro* and *in vivo*[44,45]. In our study, we observed both M2-like microglial activation and upregulation of IL-4 expression in the mice treated with HUMSC-NCs, which then could lead to the increase of NEP and IDE expression. We observed an elevation of both mRNA and protein levels of NEP and IDE by HUMSC-NC transplantation. Our results suggest that HUMSC-NC transplantation reduces Aβ deposit by elevating Aβ degradation.

M2-like microglial activation is stimulated in an AD mouse model when MSCs are transplanted into the mice [8]. In our study, single intracerebral injection of neuron-like cells differentiated from HUMSCs into a similar AD mouse model also significantly promoted M2-like microglial activation. M2-like microglia have been shown to play protective roles in AD through the following three mechanisms: promoting Aβ clearance by directly secreting enzymes, phagocytizing Aβ, and secreting neuroprotective cytokines [8,46-48].

The mechanism underlying M2-like microglial activation by cell transplantation remains unclear. It could be an indirect effect from transplanted cells. We proposed that D609-induced neuron-like cells could stimulate the secretion of some bioactive paracrine factors, which then promote M2-like microglial activation. It has also been shown that M2-like microglia can be recruited from bone marrow in an AD mouse model [7,49,50]. Thus, in our study, the source of M2-like microglia activated by HUMSC-NC transplantation could be either from bone marrow recruitment or from local resting microglia that could be activated into M2-like microglia by paracrine factors stimulated by HUMSC-NC transplantation, such as IL-4.

Antiinflammatory cytokine IL-4 has been found to promote M2-like microglial activation [51] and induces Aβ removal both *in vitro* and *in vivo*[44,45,52]. We observed an upregulation of IL-4 expression by HUMSC-NC transplantation, and our immunofluorescence staining showed that IL-4 expression was markedly increased in Iba-1-positive cells in particular. Our results suggest that M2-like microglial activation in the mice treated with HUMSC-NCs could be mediated by upregulation of IL-4 expression.

Both our RT-PCR and immunocytochemistry staining results suggest that HUMSC-NC transplantation downregulates the expression of proinflammatory cytokines, including TNF-α and IL-1β. TNF-α, IL-6, and IL-1β are markers for classic microglia (M1-like microglia). These proinflammatory cytokines have been shown to promote Aβ production and reduce Aβ removal in an AD mouse model [53]. Aβ-induced phagocytosis of microglia can be inhibited by proinflammatory cytokines, such as IL-1β, TNF-α, IFN-γ, MCP-1, and CD40L [54]. Thus, reducing these proinflammatory cytokines could promote Aβ clearance in AD. Our results suggest that HUMSC-NC transplantation reduces the chronic inflammation mediated by M1-like microglia and induces M2-like microglial activation to enhance Aβ removal, which consequently results in cognitive improvement in the AD mice.

In our study, HUMSC transplantation did not produce beneficial effects in the AD mice; although transplantation of MSCs has been shown to rescue memory deficits and reduce Aβ deposition in a similar AD mouse model [8]. The inconsistent observation might be because different experimental procedures for cell transplantation were used in the studies. In our study, we performed single injections of HUMSCs into mice, whereas in the studies of others, mice received double or triple injections of MSCs. In addition, it has been shown that a single injection of bone-marrow MSCs does not promote M2-like microglial activation and fails to improve the cognitive activity in an AD mouse model, whereas multiple transplantations produce beneficial effects [8]. We found that single transplantation of HUMSC-NCs improved memory and reduced Aβ deposition in the AD mice, which is consistent with others’ observation that single transplantation of neuron-like cells differentiated from MSCs produces beneficial effects in neurodegenerative diseases and spinal cord injury [9-11].

## Conclusion

Our study demonstrated that transplantation of neuron-like cells differentiated from MSCs isolated from human Wharton jelly of the umbilical cord decreased Aβ deposition and improved memory in AβPP/PS1 transgenic mice by a mechanism associated with activating M2-like microglia and modulating neuroinflammation. Transplantation of neuron-like cells differentiated from stem cells might be a promising cell therapy for AD.

## Abbreviations

AD: Alzheimer disease; Arg-1: Arginase-1; Aβ: Amyloid β- peptides; CD163: Haptoglobin/hemoglobin scavenger receptor; CTF: C-terminal fragment; D609: Tricyclodecan-9-yl-xanthogenate; ELISA: Enzyme-linked immunosorbent assay; FIZZ1: Found in inflammatory zone 1; HUMSC-NCs: Neuron-like cells from human umbilical cord mesenchymal stem cells; HUMSCs: Human mesenchymal stem cells isolated from Wharton jelly of the umbilical cord; IDE: Insulin-degrading enzyme; IL-1β: Interleukin-1β; IL-4: Interleukin-4; MAP2: Microtubule-associated protein 2; MRC1: Mannose receptors C type 1; NEP: Neprilysin; NSE: Neuron-specific enolase; PBS: Phosphate-buffered saline; TNF-α: Tumor necrosis factor-α; YM-1: Chitinase 3-like 3.

## Competing interests

The authors declare that they have no competing interests.

## Authors’ contributions

HY designed and performed the experiments and wrote the manuscript. ZHX participated in designing the experiments. LFW, HNY, and SNY provided assistance for data analysis, mouse-injection experiments, and ELISA assay, respectively. ZYZ, PW, and CPZ were responsible for mouse-behavior observation. JZB participated in designing the experiments and drafting the manuscript. All authors read and approved the manuscript for publication.

## Supplementary Material

Additional file 1: Figure S1 Immunocytochemistry staining for GFAP in HUMSC-NCs and positive control (astrocytes). Cells were fixed and stained with rabbit anti-human GFAP IgG (1:200). Fluorescent dye conjugated secondary antibody, goat anti-rabbit IgG-TRITC, was used to visualize the cells. The images were captured by a camera system connected to a fluorescence microscope (Olympus 1 × 71S1F-3). Click here for file
