# Supplementary material for: Human umbilical cord mesenchymal stem cell-derived neuron-like cells rescue memory deficits and reduce amyloid-beta deposition in an AβPP/PS1 transgenic mouse model
Source: Stem Cell Res Ther. 2013 Jul 4;4(4):76. doi: 10.1186/scrt227 (PMC3854736; doi:10.1186/scrt227)
Supplement: Additional file 1: Figure S1 — Immunocytochemistry staining for GFAP in HUMSC-NCs and positive control (astrocytes). Cells were fixed and stained with rabbit anti-human GFAP IgG (1:200). Fluorescent dye conjugated secondary antibody, goat anti-rabbit IgG-TRITC, was used to visualize the cells. The images were captured by a camera system connected to a fluorescence microscope (Olympus 1 × 71S1F-3). [file scrt227-S1.pdf]

Supplementary Figure 1

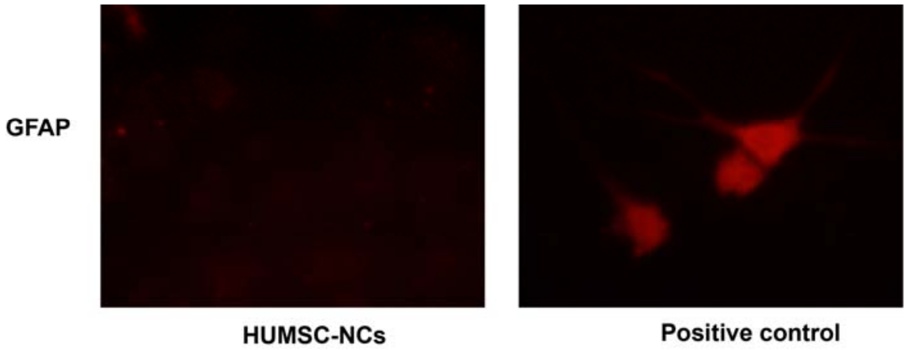

Supplementary Figure 1. Immunocytochemistry staining for GFAP in HUMSC-NCs and positive control (astrocytes). Cells were fixed and stained with rabbit anti human GFAP IgG (1:200). Fluorescent dye conjugated secondary antibody, goat anti-rabbit IgG-TRITC, was used to visualize the cells. The images were captured by a camera system connected to a fluorescence microscopy (Olympus 1x71S1F-3, JAPAN).
